# Molecular Clues to Understanding Causes of Human-Assisted Reproduction Treatment Failures and Possible Treatment Options

**DOI:** 10.3390/ijms231810357

**Published:** 2022-09-08

**Authors:** Jan Tesarik, Raquel Mendoza-Tesarik

**Affiliations:** MARGen Clinic, 18006 Granada, Spain

**Keywords:** sperm DNA damage, sperm epigenetic abnormalities, oocyte aneuploidy, oocyte cytoplasmic defects, fertilization failure, embryo cleavage arrest, implantation failure, early miscarriage, luteal phase, luteoplacental shift

## Abstract

More than forty years after the first birth following in vitro fertilization (IVF), the success rates of IVF and of IVF-derived assisted reproduction techniques (ART) still remain relatively low. Interindividual differences between infertile couples and the nature of the problems underlying their infertility appear to be underestimated nowadays. Consequently, the molecular basis of each couple’s reproductive function and of its disturbances is needed to offer an individualized diagnostic and therapeutic approaches to each couple, instead of applying a standard or minimally adapted protocols to everybody. Interindividual differences include sperm and oocyte function and health status, early (preimplantation) embryonic development, the optimal window of uterine receptivity for the implanting embryo, the function of the corpus luteum as the main source of progesterone production during the first days of pregnancy, the timing of the subsequent luteoplacental shift in progesterone production, and aberrant reactions of the uterine immune cells to the implanting and recently implanted embryos. In this article, the molecular basis that underlies each of these abnormalities is reviewed and discussed, with the aim to design specific treatment options to be used for each of them.

## 1. Introduction

Despite the fact that the first successful in-vitro fertilization (IVF) attempt dates back more than 40 years ago [1], and many further clinical and laboratory procedures related to IVF have been introduced to clinical medicine since then [2], the success rates of all these assisted reproduction techniques (ART) still remain relatively low nowadays. Published regional registry data from governments and/or specialty societies, covering the USA, Canada, the UK, Australia/New Zealand (combined), Latin America (as a block) and Japan, show clearly that the birth rate per fresh ART cycle began to decrease worldwide in 2010 [3]. In fact, it dropped from 30% in 2010 to 22% in 2016 and, according to several preliminary regional statistics, this trend still continues [3]. In addition to intrinsic factors, there also are a number of environmental influences that act as aggravating factors, notably temperature and climate change, pollutants (cars, industrial, plastics), and others. However, increasing maternal age, associated with various oocyte molecular abnormalities, appears to be the main culprit [4,5,6].

Despite the fact that, over the past 40 years, ART techniques have undergone continuous expansion, incorporating new methodological innovations, their basic steps remain the same [2]. Briefly, oocytes are recovered by puncture and aspiration of large ovarian follicles after hormonal stimulation, with preparations containing follicle stimulating hormone (FSH) and luteinizing hormone (LH) activity and the induction of final oocyte maturation with human chorionic gonadotropin (hCG). Subsequently, the oocytes recovered are fertilized in vitro, sometimes by using micromanipulation techniques, such as intracytoplasmic sperm injection (ICSI) [7,8], with spermatozoa obtained from the ejaculate or by testicular biopsy. After 2–5 days of in vitro culture, the resulting embryos are transferred to the uterus.

Over the past few years, there have been more and more voices that campaign for the introduction of new strategies in order to improve the overall ART outcomes at the global level. Most of these voices agree on the need for abandoning the generalized use of routine or minimally adapted clinical and laboratory techniques in all infertile couples and adopting a personalized approach, based on molecular analysis of each individual case [4,5,6].

There are quite a few recent studies that deal with the different molecular mechanisms involved in the processes of gamete development and maturation, fertilization, pre-implantation embryo development, implantation and early post-implantation development. However, there is still a paucity of information about the links between the abnormalities of these mechanisms and a specific treatment to be used in the clinical setting.

This short review summarizes the most significant recent findings related to the molecular events required for normal gamete development, fertilization, the early embryonic development, and uterine receptivity for the implanting embryo. These data can serve as a guide for future studies aimed at designing new diagnostic and therapeutic methods with which to improve the current efficacy of ART.

## 2. Impaired Sperm Function

After the introduction of micromanipulation techniques, namely intracytoplasmic sperm injection (ICSI) to assist fertilization [7,8], many diagnostic and treatment methods, aimed at improving the outcomes of conventional IVF by assisting sperm interaction with the zona pelucida and sperm-oocyte fusion, lost their importance in ART, even though they can still be helpful to determine the cause of unexplained infertility. However, despite the initial hopes that ICSI would be able to resolve all cases of male infertility, provided that spermatozoa can be recovered, subsequent studies showed that fertilization can fail even if a spermatozoon is successfully placed in the oocyte cytoplasm. Failure of oocyte activation by the injected spermatozoon, skipping the cell-signaling pathways activated by the early contact of the fertilizing spermatozoon with the oocyte plasma membrane, was the first obstacle to be addressed [9]. This problem can be caused by a deficiency of oocyte-activating factors in the injected spermatozoon or by the failure of oocytes to respond correctly to these factors. This issue is practically resolved nowadays by the use of different methods of artificial oocyte activation [10,11,12].

**Figure 1:**
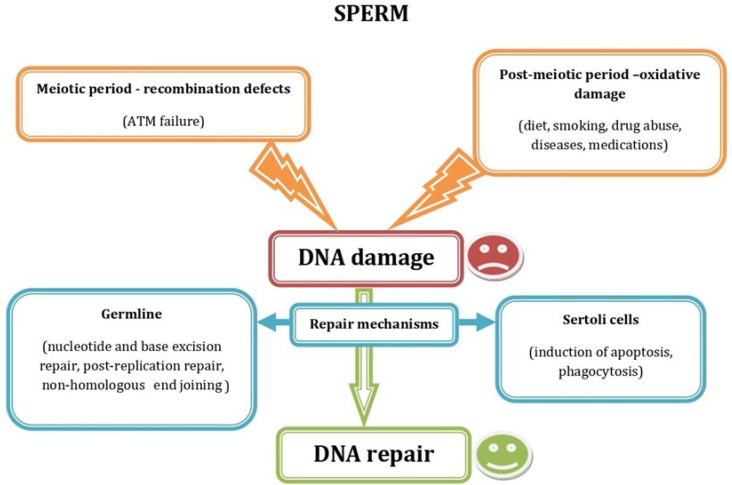
Schematic representation showing the most common factors causing sperm DNA damage during the meiotic and the postmeiotic period and the DNA repair mechanisms acting in germ and Sertoli cells.

However, once the problem of oocyte activation had been resolved, a new serious obstacle, sperm DNA fragmentation, made its appearance. Sperm DNA fragmentation is caused by distinct mechanisms (Figure 1) and can involve only one of the two complementary strands that form the DNA double helix, thus producing single-strand breaks (SSB), or both of them, resulting in double-strand breaks (DSB). This kind of DNA damage is more common in spermatozoa with overall chromatin structure abnormalities (protamination, compaction, etc.). Sperm aneuploidies are also very relevant for fertilization success. However, unlike oocyte aneuploidies, they do not appear to be related to paternal age [13]. It is well-known that factors causing DNA fragmentation, such as dietary preferences, smoking, chronic alcoholism, drug addiction, and various diseases and drug therapies (reviewed in [13]), mostly act via an excess of reactive oxygen species [14]. However, sperm DNA fragmentation can also be caused by a different mechanism, related to DNA recombination between homologous chromosomes during meiosis (Figure 1). In fact, primary spermatocytes deliberately produce DSB of their DNA molecules, as a physiological process in prophase of the first meiotic division, to allow DNA recombination between homologous chromosomes [15]. After recombination, the protein kinase called ataxia-telangiectasia mutated (ATM) activates the mechanisms that repair the free ends to generate the chiasma and prevent the formation of new DSB, once the recombination process is accomplished [16]. If these mechanisms fail, DSB will persist until the stage of mature spermatozoon.

Repair of both SSB and DSB can occur later, in the testicular germinal epithelium or in the zygote after fertilization [17]. Different DNA repair mechanisms (Figure 1), including nucleotide excision repair, base excision repair, DNA mismatch repair, DSB repair, post-replication repair and non-homologous end joining, are functional in the human germline [18]. The repair capacity of the testicular germinal epithelium appears to be related to the correct function of Sertoli cells that participate in apoptosis of DNA-damaged germ cells and remove them by phagocytosis [19,20]. If all of these repair and selection mechanisms fail, spermatozoa with damaged DNA reach the ejaculate and, when introduced to the oocyte, can disturb subsequent embryo development by different mechanisms (see setions 3 and 4 of this article), although some remaining sperm-derived DNA damage can still be repaired within the oocyte. It has been suggested that the oocyte has the capacity to repair sperm DNA damage when the level of sperm DNA damage is less than 8% [18].

In addition to sperm DNA fragmentation, mutations or abnormal expression of specific genes in male spermatogenic cells cause subsequent problems of embryo development, which is also the case for epigenetic disorders [13]. Many of these problems are related to aberrant expression of the genes involved in the regulation of estrogen signaling. Alterations of estrogen receptor β gene RsaI polymorphism, contributing both to a reduced fertilization rate and impaired embryonic developmental competence [21], is an example of this pathogenetic mechanism. Inadequate protein expression level of the highly evolutionary conserved sperm post-acrosomal region WW-domain binding protein (PAWP) leads to abnormal calcium signaling in response to the fertilizing spermatozoon [22], resulting in abnormal postfertilization development [23]. Sperm aneuploidies are also very relevant for fertilization success [13].

## 3. Impaired Oocyte Function

Aneuploid conception, resulting from chromosome segregation errors during the meiotic divisions in human female meiosis, is the main oocyte-derived cause of ART failure [24]. Oocyte aneuploidies are related to increasing maternal age [25,26] and, traditionally, they have been attributed to oxidative stress and defective functions of oocyte mitochondria [27]. However, recent observations show that oxidative stress is not the only factor underlying oocyte aneuploidy, and other factors, also present in some oocytes from young women, enter into play.

**Figure 2:**
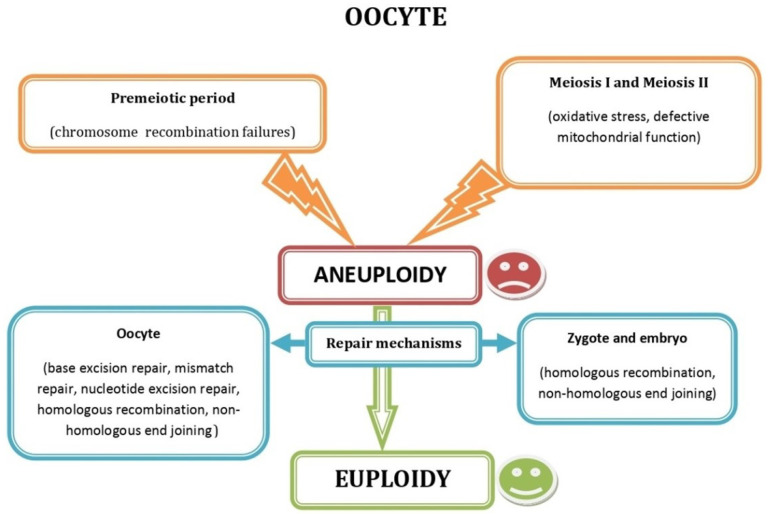
Schematic representation showing the most common factors causing oocyte aneuploidy during the premeiotic and the meiotic period and the DNA repair mechanisms acting in oocytes, zygotes and embryos.

Analysis of genome-wide maps of recombination and chromosome segregation in human oocytes uncovered a new reverse chromosome segregation pattern concerning sister chromatid separation at meiosis I, influencing the elimination of aneuploid embryos and is responsible for the chromosomal drive against non-recombinant chromatids at meiosis II [28]. Moreover, in addition to the errors in meiosis I or II of oogenesis [29], oocyte autosomal aneuploidy can also arise from germinal mosaicism, leading to premeiotic aneuploidy (Figure 2) that affects at least 10% of unselected oocytes irrespective of maternal age and may, thus, account for some cases of aneuploid conceptions in very young women [30].

It was shown recently that the propensity of individual chromosomes to meiotic errors is closely related to the number of crossovers (interchromatid recombination sites between chromatids of homologous chromosomes), forming synaptonemal complexes during the first meiotic prophase, chromosomes and oocytes with fewer crossovers being more susceptible to chromosome segregation errors [31,32]. Failure of homologous chromosomes to recombine is a common feature of human oogenesis, and women with lower rates of meiotic recombination are at an increased risk of producing aneuploid embryos [33].

Even though oocyte aneuploidies are responsible for most ART failures, especially in women of advanced age, other oocyte-borne ART failures are caused by specific gene variations, methylation pattern abnormalities, and inadequate gene expression levels (reviewed in Sfakianoudis et al. [34]).

The age-related diminished ovarian reserve issue can hardly be resolved. However, it is rarely responsible for oocyte quality. The quality of oocytes is mainly dependent on the female age, and it can be ameliorated, to some extent, by medical treatments in most cases [34].

Embryonic development is particularly sensitive to differential expression or mutation of numerous genes that control the pathways involved in cell cycle regulation, DNA repair and oocyte cytoplasmic maturation [34].

Because of the relatively late onset of embryonic gene expression in humans and some other mammalian species (see Section 4), the accumulation of maternal RNA and protein molecules, needed during early human preimplantation development, in the oocyte cytoplasm makes the oocyte quite different from somatic cells, where RNA and proteins usually undergo rapid turnover [35]. Maternal mRNAs are translationally silenced by cytoplasmic polyadenylation machinery, which is also responsible for their timely translational activation during post-fertilization development [36]. Other cytoplasmic components, such as non-coding regulatory RNAs, proteins and mitochondria of oocyte origin, are also important for future embryo development. In particular, oocyte mitochondrial dysfunction is known to be responsible for embryo abnormalities [37]. Mitochondrial DNA mutations are frequent, but only a few of them are related to adult mitochondrial disease [38].

## 4. Impaired Preimplantation Embryo Development

**Figure 3:**
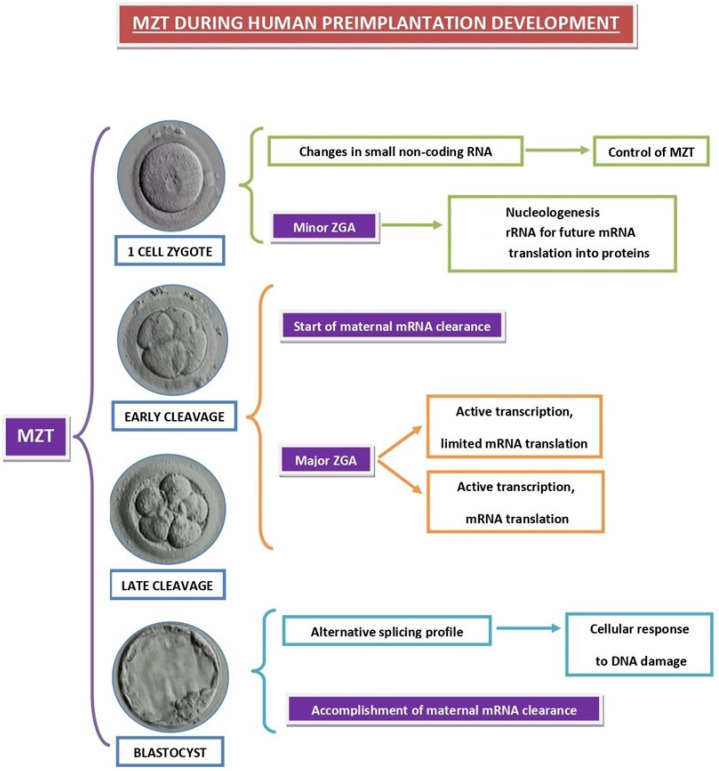
The main events that occur during MZT in human preimplantation embryos.

Preimplantation embryo development encompasses the period between fertilization and embryo implantation in the uterus. Maternal-to-zygotic transition (MZT), during which transcripts of maternal genes are removed by degradation and the zygotic genome activation (ZGA) occurs (Figure 3), is a critical event during this period in all animal species [39,40]. MZT failure invariably leads to embryo developmental failure, so that precise control over the time of ZGA and maternal transcript degradation is essential for normal embryogenesis [41].

ZGA was shown recently to occur in two waves, both in mice [42] and in humans [43]. While a major ZGA occurs at the 2-cell stage in mice [42] and between the 4-cell and 8-cell stage in humans [44,45,46], it is preceded by minor gene activity that is detected as early as the 1-cell stage in both species [41,42]. Transciption was detected as early as the 4-cell stage in human embryos [44], while signs of embryonic gene expression, revealed by the appearance of specific proteins [45] and quantitave submicroscopical changes in embryonic organelles [46], mostly appear at the 6- to 8-cell stage. Minor gene transcriptional activity in human 1-cell and 2-cell embryos was localized by high-resolution autoradiography to perinucleolar chromatin regions and suggested to be involved in the transformation of nucleolar precursor bodies into nucleoli [47,48]. In fact, this limited RNA synthesis in human 1-cell zygotes (Figure 3) is critical for nucleolar assembly [48].

In addition to nucleologenesis, the early wave of human zygotic transcription also accounts for changes in the profile of small noncoding RNAs (sRNAs) that play important roles during MZT [49]. Altogether, these data indicate that only few, if any, RNA molecules synthesized during the initial minor wave of ZGA are translated into proteins, and these RNAs act directly to ensure the vital needs of the developing embryo, such as the nucleologenesis required for subsequent mRNA translation in ribosomes, and the creation of the adequate profile of sRNAs to coordinate the proceses of embryonic gene transcription and maternal mRNA decay that takes place during the upcoming major wave of ZGA.

In view of the above consideration, preimplantation embryo development can be considered the main period during which the fate of the embryo unfolds, for better or worse. With respect to the timing of MZT, it can be subdivided into the following three main phases: pre-MZT, major ZGA, and accomplished MZT.

### 4.1. Pre-MZT Phase

Before the major ZGA, which occurs between the 4-cell and 8-cell stage of embryonic development in humans (see above), the developmental processes that take place in the embryonic cells are mainly under maternal control. During this period of transcriptional silence, development is driven by cytoplasmic factors, which largely consist of maternally deposited mRNAs, whose proper function requires strict temporal and spatial regulation [36]. Studies performed in the mouse model show that, in the absence of transcriptional input from the zygotic genome, embryos necessitate the regulation of maternal mRNAs at the post-transcriptional level in order to activate different subsets of mRNAs with precise developmental timing; this translational control is mediated by changes in maternal mRNA polyadenylation [50,51].

A recent study [52] analyzed the association between maternal effect genes, encoding mRNA and other factors that control embryonic development prior to the activation of the embryonic genome in human oocytes, on the one hand, and late developmental abnormalities and birth defects on the other hand. Over 80 maternal effect genes were identified, and their variations were shown to be associated with a range of adverse outcomes in humans, including zygotic cleavage failure, hydatidiform moles, multi-locus imprinting disorders in the offspring, and congenital heart defects [52]. These findings underscore the importance of the pre-MZT phase, whose irregularities affect not only the immediate subsequent steps of embryo development, but also may cause long-term effects, conditioning offspring health.

### 4.2. Major ZGA Phase

Even though the timing of the major ZGA activation in different mammalian species, including humans, has been known since the 1980s [41,44,45,46], the molecular mechanisms that control this event were largely unknown until recently [34,49,53]. Studies performed in different animal species indicate that ZGA can be controlled by a number of factors, including cell cycle length, transcriptional repressors and activators, and changes in chromatin organization, although the relative importance of each of these factors varies among species [54]. In humans, preimplantation embryogenesis is a remarkably complicated, well-orchestrated process that relies on synchronization of oocyte maturation and ZGA, and it appears that the translational program of maternal mRNAs plays a key-role in human ZGA [55]. In recent studies, double homeobox 4 (DUX4) was identified as a multifunctional factor that primes human embryonic genome activation [53,56,57]. In fact, DUX4 is expressed in early human embryos and the DUX4 binding motif is enriched in the promoter regions of the human genes involved in ZGA, such as LEUTX (at the 4-cell stage), and ARGFX, DPRX and TPRX (at the 8-cell stage) [56,57].

Transcriptomic data from human, mouse, and cow preimplantation development also show that the period of ZGA exhibits the highest level of mRNA alternative splicing and most of the exon skipping occurs in the genes responsible for DNA damage response [58]. It was suggested that developmentally programmed splicing failure at ZGA contributes to the attenuation of p53-mediated cellular responses to DNA damage [58].

### 4.3. Accomplished MZT Phase

Progressive elimination of stored maternal mRNAs is required for MZT to be accomplished [39,59]. Transcriptome analyses indicated that maternal mRNA clearance is largely completed by the 8-cell stage of human embryos [60,61]. Unlike its involvement in mice, zygotic transcription plays a more important role in the decay of human maternal transcripts, so that the duration from ZGA to the completion of maternal mRNA elimination is longer in humans compared to that in mice [59]. In addition, mRNAs of both the maternal and the zygotic origin are likely to be simultaneously translated in human embryos for a short time before the MZT is definitively accomplished. This was suggested by a qualitative and quantitative electron microscopic study combined with autoradiography, showing that modifications of the plasma membrane at the intercellular contact regions required for the subsequent formation of the blastocyst cavity are the same in embryos that have properly enhanced their transcriptional activity at the 8-cell stage and in those that have not [62]. However, extended persistence of maternal transcripts after ZGA is detrimental to further embryonic development [59].

Cytoplasmic polyadenylation-mediated translational control of maternal mRNAs directs ZGA and MZT in many mammalian and non-mammalian species [63]. However, there are interspecies differences regarding the molecular mechanisms of these processes [63], and data concerning human embryos have only started to emerge recently. The elimination of maternal mRNA after ZGA in human embryos is mediated by a variety of mechanisms, involving changes in the expression of regulatory molecules, including YAP1-TEAD4 transcription activators, TUT4/7-mediated mRNA 3′-oligouridylation, and BTG4/CCR4-NOT-induced mRNA deadenylation, since decreased expression of these factors was observed in development-arrested embryos of patients undergoing ART attempts [59]. These events appear to be orchestrated by changes in the profile of small non-coding RNAs (sRNAs) in the embryos, showing a shift from the prevalence of oocyte-derived short Piwi-interacting RNAs (piRNAs) to increasing expression of microRNA (miRNA) in cleavage-stage human embryos [49]. Moreover, data on small RNA sequencing of spent culture media from morula-stage human embryos, showing different development potential and implantation success [64], provide a view of a more complex network that controls human preimplantation development.

Future work is needed to understand the mechanisms that regulate the reprogramming of the maternal sRNA profile, as well as confirming whether, when, and how miRNAs function during early embryonic development in humans. A systematic review and critical analysis of published data about molecular drivers of developmental arrest in the human preimplantation embryo have been published recently [34] and will hopefully lead to designing research focused on this field in the future.

## 5. Impaired Uterine Receptivity

Uterine receptivity refers to a condition in which the uterus is suitable for embryo implantation, and it lasts for a limited time, which is defined as the implantation window. The concepts of uterine receptivity and implantation window were established in the 1960s, based on studies that employed the embryo transfer technique in different animal species (reviewed in Tu et al. [65]). In humans, the first 7 days of the secretory phase is considered as the pre-receptive stage, 7–10 days after ovulation as the receptive stage (implantation window) and the rest of the secretory phase is defined as the non-receptive stage [66,67,68]. Uterine receptivity is governed by comples molecular mechanisms, showing significant interspecies differences. Most data were obtained by animal experiments, while the knowledge in humans is still limited.

In simple terms, the implantation window in all species studied so far is opened and closed by first messengers (mainly hormones,) whose action activates second messengers and downstream signal transduction pathways. Abnormalities at any of these levels can cause recurrent implantation failure (RIF) in humans, but the critical underlying molecular mechanisms still remain largely unknown [69].

### 5.1. First Messenger Failure

First messenger abnormalities responsible for embryo implantation failure have been most studied and represent a relatively easy target for treatment action, based on the concept of substitutive therapy. Estrogen and progesterone are the main first messengers responsible for the preparation of the uterus for embryo implantation [70]. Estrogen and progesterone act mainly through nuclear receptors, including estrogen receptors (ER) and progesterone receptors (PR), respectively. Both ER and PR are ligand-dependent nuclear transcription factors and have complex crosstalk with other signaling pathways [65]. While progesterone is necessary for implantation in almost all mammalian species, including humans, some species do not need estrogen, and the estrogen function in non-human primates and the human remains inconclusive [71]. Even though estradiol is known to interact coordinately with progesterone in endometrial development, excess estradiol levels on the day of progesterone treatment initiation were suggested to negatively affect pregnancy outcomes during artificial cycles for frozen cleaved embryo transfer, whereas such an effect was not observed for blastocyst transfer [72].

Supraphysiological estrogen and suboptimal progesterone levels are common in ART attempts using different ovarian stimulation protocols and ovulation triggers, causing the condition known as luteal phase deficiency (LPD) [73]. Low progesterone levels resulting from LPD not only disturb the process of decidualization, but also lead to dysfunction of the local uterine immune system, with an increased risk of embryo rejection, abnormally high uterine contractility, and restriction of uterine blood flow [73].

In addition to progesterone, growth hormone (GH) is another first messenger implicated in the acquisition of the optimal uterine receptivity in humans. This was first unequivocally demonstrated by a randomized controlled trial (RCT) that involved women with RIF after the transfer of embryos in an oocyte donation program, where oocytes were recovered from young donors not treated with GH [74]. The RIF patients that received GH showed a significantly thicker endometrium and higher pregnancy and live birth rates as compared with RIF patients of the non-GH study group, although these rates still remained somewhat lower as compared with the non-RIF patients of the positive control group [74]. Both GH and GH receptor (GHR) are expressed in the uterus of several mammalian species, including the human (reviewed in Liu et al. [75]). In addition to GHR, some of the GH effects in the uterus appear to be mediated by GH-induced insulin-like growth factor (IGF) 1 expression, since sequential activation of uterine epithelial IGF1R by stromal IGF1 and embryonic IGF2 was shown to direct normal uterine preparation for embryo implantation in mice [76]. As for the human uterus, however, the exact mechanism of GH action still remains to be elucidated.

A number of other, non-hormonal ligands were also shown to affect uterine receptivity in animal models. These include leukemia inhibiting factor (LIF) [77], bone morphogenetic protein 2 (Bmp2) [78,79], osteopontin [80], different cytokines and chemokines [81], integrins [82,83], and epidermal growth factor (EGF) family growth factors [84]. Even though abnormal expression of some of them, namely integrins [85], selectins [86], and LIF [87,88], were suggested to be associated with RIF in humans, the data are not conclusive, and the subject of the role of non-hormonal ligands in the regulation of human uterine receptivity is still understudied.

### 5.2. Failure of Post-Receptor Signal Transduction Pathways

Both estradiol and progesterone, acting sequentially as the main drivers of uterine receptivity, bind to their nuclear receptors, acting as transcription factors through estradiol and progesterone response elements, respectively. The action of both hormones affects numerous post-receptor cell signaling elements, including growth factors, transcription factors, lipid mediators, cytokines, and cell cycle regulators involved in the initiation of pregnancy [66]. In the rat model, progesterone receptor (PR) and estrogen receptor (ER) were shown to control the early decidualization steps through concomitant regulation of heart- and neural crest derivatives-expressed protein 2 (Hand2), Bmp2, and extracellular signal-regulated kinases 1 and 2 (ERK1/2) [89]. The transcription factors involved in the regulation of uterine receptivity include products of homeobox (Hox), containing family genes. Two of them, Hoxa10 and Hoxa11, have been demonstrated to be necessary for uterine receptivity and implantation in mice, and recent evidence suggested these factors play a similar role in humans [90]. As for the other post-receptor cell signaling elements that have been shown to mediate estradiol and progesterone action in rats and mice, information about their action in humans remains to be demonstrated.

Unlike estradiol and progesterone, GHR is located on the cell surface, and upon ligand binding, it activates the Janus kinase (JAK)-signal transducer and activator of the transcription (STAT) signaling pathway, in addition to activating the Src family kinase signaling pathway independent of JAK [91]. Interestingly, STAT3 signaling also mediates the transduction of the LIF receptor (LIFR)-generated signal to downstream the effectors [92]. Because a low level of LIF is associated with unexplained miscarriages and RIF in humans [87,88], it can be hypothesized that GH can partly compensate for LIF insufficiency.

## 6. Pregnancy Failure after Implantation Stage

Among the different factors responsible for pregnancy loss after implantation, diminished production of progesterone is particularly frequent after ART treatments, although it can also occur after natural conception [73]. At the beginning of pregnancy, progesterone is almost exclusively secreted by the corpus luteum, stimulated by endogenous hCG from the implanted embryo. However, even in the case of continuous stimulation by endonenous hCG, the lifespan of the corpus luteum does not exceed several weeks, and during this time, its production of progesterone wanes progressively, beginning as early as the first month of pregnancy [73]. However, at the same time, progesterone production shifts progressively from the corpus luteum to the placenta, thus compensating for the diminished progesterone secretion by the corpus luteum. This phenomenon is referred to as the luteoplacental shift (LPS). After complete luteolysis, the placenta becomes the main source of progesterone. It is, thus, evident that the waning of the corpus luteum and the increase in placental progesterone production must be correlated so as to ensure normal circulating progesterone levels. However, this is not always the case and luteolysis can proceed more rapidly than the activation of placental progesterone production, a condition that mostly leads to the loss of pregnancy [93].

## 7. Clues to Possible Treatment Options

### 7.1. Male Infertility

Despite the existence of a plethora of data related to the different molecular events that control fertilization, preimplantation embryo development and implantation, only few of them are at the origin of specific molecular-based therapies. This is partly due to the fact that most of these data stem from experiments carried out in animals, mainly rodents, while experiments in humans are restricted by ethical concerns. Unfortunately, with the exception of non-human primates, the mechanisms of early embryo formation, development and implantation show significant differences between experimental animals, and rodents in particular, on the one hand, and the human on the other hand. Hopefully, increasing knowledge of the abnormalities responsible for early developmental failures in the human will make it possible to design molecular treatment strategies, based on genome editing, in the future. However, despite enormous pressure by infertile couples whose problems cannot be solved by conventional methods, experts warn against the use of human germline genome editing, which may cause serious problems to the offspring; thus, all related techniques should be handled with care [94]. Nevertheless, some treatment options based on molecular data are already available in human ART.

After the advent of ICSI, many sperm abnormalities, leading to defective sperm movement, sperm-zona pellucida attachment, acrosome reaction and zona pellucida penetration, and sperm-oocyte fusion, can be easily overcome by ART. Hence, sperm DNA damage and deficiency of oocyte-activating factors remain the only serious sperm defects that still can cause ART failure in the ICSI era. Assisted oocyte activation (AOA), with the use of calcium ionophores [95], or mechanical manipulation during ICSI [11] resolves the issue of spontaneous oocyte activation failure in most cases. A recent systematic review and meta-analysis shows that, in addition to increasing fertilization rates, AOA with calcium ionophores also improves pregnancy outcomes and offspring safety in infertile patients [96].

Sperm DNA damage can sometimes be mitigated by changing diet and lifestyle and by eliminating its primary cause, such as varicocele or infection, if it can be determined [97]. However, when no specific cause is identified or if the above measures fail, recourse to non-specific treatments has to be considered. There are two types of treatments available, one aimed at the reduction in the proportion of spermatozoa carrying damaged DNA and the other using different laboratory methods to select healthy spermatozoa in vitro for assisted reproduction. Oral treatment with different antioxidants, used alone or in combination, is the most common approach, aimed at reducing sperm DNA damage. In 2005, a randomized controlled trial showed that oral treatment with 1 g vitamin C and 1 g vitamin E daily for 2 months significantly reduced the percentage of DNA-fragmented spermatozoa [98]. Later studies confirmed this result and extended the range of orally administered antioxidants, including L-carnitine, L-acetyl carnitine, coenzyme Q10, selenium, and lycopene. Either administered alone or in combination, these antioxidants were reported to enhance semen parameters and sperm DNA integrity in idiopathic infertile men [99,100]. However, caution is needed to choose an adequate dose of the antioxidants used, since excess antioxidants can shift the cellular redox balance from oxidative stress to an opposite extreme called reductive stress, which is not only counterproductive in male infertility treatment, but can also negatively influence the general health status by increasing the propensity to diseases such as cancer and cardiomyophathy [101]. The adjustment of the types and doses of the antioxidants used can be finely tuned by determining the seminal oxidation–reduction potential, which provides the overall balance between oxidants and antioxidants measured by MiOXSYS [102,103].

The other treatment strategy is based on the selection of spermatozoa with intact DNA by different laboratory techniques and is used in the context of ART. The techniques reported to be capable of selection against spermatozoa with damaged DNA, so as to facilitate the use of healthy spermatozoa for ART, include physiologic ICSI (PICSI), based on the capacity of intact spermatozoa to bind to hyaluronic acid, magnetic-activated cell sorting (MACS) [104], intracytoplasmic morphologically selected sperm injection (IMSI) using high-resolution microscopy to pick up morphologically normal spermatozoa [105], and ICSI with spermatozoa recovered by testicular sperm extraction (TESE-ICSI) [106,107]. An algorithm for choosing each of the above methods, used alone or in combination, according to the severity of the patient’s condition, has been suggested [108].

### 7.2. Female Infertility

As is the case with male infertility, antioxidant treatment is also indicated in most cases of female infertility (Table 1), especially in those related to both age-related and unrelated ovarian factors [109]. The treatment protocols used involve both oral treatment and in vitro supplementation of antioxidants via media for gamete and embryo culture (only applicable in case ART techniques are used). A wide range of different antioxidants were tested, including direct reactive oxygen species (ROS) scavengers, such as vitamins C and E, substances that act indirectly by stimulating intracelular signaling pathways, leading to increased activation of endogenous antioxidant systems, such as growth hormones, and those combining both of these activities, such as melatonin [27].

In young women, antioxidants were mainly used in the following two clinical indications: polycystic ovary syndrome (PCOS) and endometriosis. In women with PCOS, treatment with resveratrol improved IVF outcomes, probably by changing the serum levels of some sex hormones and expression of VEGF and HIF1 genes involved in the angiogenesis pathway of granulosa cells [110]. Improvement of clinical outcomes of IVF treatment in PCOS women was also reported with the use of growth hormone (GH) [111], vitamins D and E [112], and an antioxidant mixture, consisting of vitamins A, B2, B6, B12, C, D3, nicotinamide, and folic acid [113]. As for patients with endometriosis, different antioxidants have been shown to reduce endometriotic lesions and alleviate pain, but no promising effect on fertility improvement was reported [114].

In both young and older women, the main cell-signaling pathways involved in age-related or unrelated ovarian failure are those that control cell protection against oxidative stress [27]. A single-cell transcriptomic analysis of ovaries from young and aged non-human primates identified seven ovarian cell types with distinct gene-expression signatures and pointed out disturbances in antioxidant signaling in early-stage oocytes and granulosa cells [115]. Based on these observations, different types of antioxidants, such as coenzyme Q10 [116,117,118], vitamin C, vitamin E and folic acid [119], have been used to improve oocyte quality and quantity (Table 1).

However, the first substance proven to increase ongoing pregnancy and live birth rates in humans in a randomized controlled trial (RCT), published in 2005, was GH [120]. Subsequent studies [121,122,123,124,125,126], including one meta-analysis of RCTs [127], confirmed these observations but also suggested a need for more RCTs with large sample sizes to elucidate the mechanism of GH action and to distinguish between patients who would benefit from GH treatment from those who would not [125]. A recent study showing that “GH age”, determined by measuring serum concentrations of IGF-1, is much higher as compared to chronological age in those patients who respond positively to GH treatment [128], marks a step in this direction. GH has also been shown to improve ART outcomes in young women with recurrent implantation failure (RIF) and early abortion [129] and in those suffering from polycystic ovary syndrome (PCOS), regardless of age [111].

The exact mechanism of GH action in the human ovary still remains to be determined. Even though it is tempting to speculate that GH influences the signaling pathways that regulate intracelular redox balance, there is no experimental finding in favor of this hypothesis, and other possible mechanisms also have to be considered. For instance, GH co-treatment during ovarian stimulation for IVF in older women increases the density of receptors for FSH, LH and BMPR1B, as well as of its own receptors, in granulosa cells [130].

The action of FSH and LH in this category of patients can also be enhanced by oral treatment with pentoxifylline, which supposedly acts by increasing the intracelular levels of cyclic adenosin monophosphate (cAMP), the second messenger that transduces the FSH-and LH-generated signal to downstream the elements of the pathway [131].

As is the case with GH, melatonin is another multifaceted agent used in ART to improve oocyte and embryo quality. In 2008, a prospective study by Tamura et al. [132] showed that melatonin at the dose of 3 mg daily, taken during one month before oocyte retrieval for an IVF attempt, significantly improved fertilization and pregnancy rates, as compared with the control group in women with poor oocyte quality in a previous attempt. Subsequent studies (reviewed in [133,134]) confirmed these observations and suggested that long-term administration of melatonin can also slow down ovarian aging. In addition to its action as a direct and indirect antioxidant, melatonin also appears to have other beneficial effects that are unrelated to preventing oxidative damage. In mice, for instance, melatonin protects oocytes from DNA damage by enhancing nonhomologous end-joining repair [135], an action that still remains to be confirmed in humans (Table 1).

**Table 1:** Treatments that improve female fertility and their tentative mechanisms of action *.

Treatment	Mechanism of Action	Target Cells	Reference
ROS scavengers	Re-establishment of redox balance	Oocytes, granulosa cells	[109,116,117,118,119]
	Protection of mitochondria	Endometriotic cells	[114]
GH	Activation of endogenous antioxidant systems	Oocytes, granulosa cells	[109]
	Induction of FSHR, LHR, GHR and BMPR1B	Granulosa cells	[130]
Pentoxifylline	Increasing intracellular cAMP	Granulosa cells	[131]
Melatonin	Direct ROS scavenger	Oocytes, granulosa cells	[109,133,134]
	Activation of endogenous antioxidant systems	Oocyte, granulosa cells	[109,133,134]
	DNA damage repair	Oocytes	[135]

* Abbreviations: ROS, reactive oxygen species; FSHR, FSH receptor; LHR, LH receptor; GHR, GH receptor; BMPR1B, BMP receptor type 1B; cAMP, cyclic adenosine monophosphate.

As for impaired uterine receptivity in the context of ART, luteal phase deficiency (LPD) [73] and failure of the luteoplacental shift (LPS) of progesterone secretion [93] are the main causes. The factors responsible for these conditions are related to the protocols used for ovarian stimulation and induction of ovulation [73,135]. LPD requires frequent checks of serum progesterone concentration [73] and an individualized approach to luteal phase support [136], which can be carried out by corpus luteum stimulation using preparations with LH activity, by direct administration of progesterone or by a combination of both [73,136]. The timing of LPS of progesterone production is also subject to strong interindividual variations with possible adverse consequences for pregnancy, including miscarriage [93,137]. It was suggested that some patients need prolonged serum progesterone checks and continuous administration of progesterone far beyond the time of gestation at which LPS normally occurs [137].

Uterine receptivity can also be disturbed by inadequate response of the endometrium to estrogen. In these cases, endometrium remains thin in the proliferative phase, despite the high doses of estrogen administered, and other adjunctive treatments, such as low-dose aspirin, pentoxifylline, tocopherol and vaginal sildenafil citrate [138], were evaluated. Following a pilot cohort study that showed encouraging results with the use of intrauterine perfusion with granulocyte colony-stimulating factor (G-CSF) to boost endometrial growth in this category of patients [139], further studies, including three meta-analyses, confirmed the beneficial effect of G-CSF both using the intrauterine and subcutaneous route of administration [140,141].

These effects of G-CSF may be related to the fact that, as is the case with GHR [91] and LIFR [92], the G-CSF receptor (G-CSFR) also activates the JAK-STAT signaling pathway [142]. Moreover, similar to ER and PR [89], G-CSFR strongly activates the MAPK/ERK pathway [143]. Hence, it is possible that the relative insufficiency of one of these agents can be partly substituted by the others at the post-receptor level. The same synergy appears to exist between GHR, G-CSFR and LIFR, on the one hand, and ER and PR on the other hand, since the JAK-STAT signaling pathway, activated by GHR, G-CSFR and LIFR, has been shown to display dynamic interactions with the HOX cascade [143,144], which is involved in ER and PR signaling [90]. However, the above suggested molecular mechanisms still remain hypothetical, and more studies are needed, especially in humans.

Other treatment methods suggested to improve endometrial thickness and receptivity, namely endometrial scratching and intrauterine injection of autologous platelet-rich plasma (PRP), intend to modulate the release of various autologous cytokines and growth factors to the uterine cavity. Endometrial scratching (local intentional injury to the endometrium) was first proposed in 2003 [145] and became quite popular thereafter. However, a recent properly powered randomized trial [146], and a meta-analysis of randomized controlled trials [147] failed to confirm the benefit of endometrial scratching before an IVF attempt, even in cases of RIF. Intrauterine injection of PRP was successfully applied for the first time in 2015 as an intervention for improving the refractory endometrium of women to receive IVF [148], and since then, promising results were reported in three randomized controlled trials (reviewed in [149]). These data have led to the suggestion to replace endometrial scratching with GH and PRP treatments as the first-line approach to RIF and early miscarriages [150].

A hostile reaction of maternal endometrial natural killer (NK) cells to HLA antigens of the embryo, which actually represents an allograft carrying HLA-C antigens of both maternal and paternal origin, also represents a possible cause of RIF. Even though, in general, maternal NK cells play an important role in placentation and postimplantation embryonic development [151], the combination of maternal killer-immunoglobulin-like receptor (KIR) AA genotype and embryonic HLA-C2 was reported to be associated with RIF, miscarriage and pre-eclampsia [152,153]. However, maternal KIR genotype evaluation in NK cells from peripheral blood mostly leads to false positive diagnostic conclusions because endometrial NK cells reveal a tissue-specific receptor repertoire, different from peripheral blood, as a result of the fine tuning of these cells towards the reception of an allogeneic embryo, and the only reliable data can be obtained in NK cells from menstrual blood [154]. This kind of analysis may confirm endometrial NK cell hostility and vaginal administration of sildenafil, a type 5-specific phosphodiesterase inhibitor, was shown to decrease NK cell hostility towards the implanting embryo and to enhance the chance of pregnancy in women with a history of recurrent miscarriage [155]. Surprisingly, lower rather than higher concentrations of pro-inflammatory cytokines were detected in women with recurrent pregnancy loss compared to fertile women, suggesting the exhaustion of the immune system, and sildenafil re-established the adequate balance between pro-inflammatory Th1 and anti-inflammatory Th2 cytokines during sequential phases of decidualization [156]. In women with luteal phase deficiency and low serum progesterone levels, adequate luteal phase support can also resolve the problem, since progesterone affects endometrial NK cells, causing them to reduce their cytotoxic functions and play a supportive role in pregnancy [73].

## 8. Conclusions

Various factors can cause human ART failures. These can be related both to sperm (DNA damage; epigenetic abnormalities, aneuploidies) and oocyte (aneuploidies, cytoplasmic defects) function, as well as to abnormal interaction between the embryo to implant and the uterine environment (immune rejection, luteal phasse abnorrmalities, delayed luteoplacental shift, etc.). This means that sperm and oocyte quality must be checked before any ART attempt, and appropriate measures must be taken against each of them. Collateral factors that potentially cause ART failure (hormonal imbalance, insulin resistance, thyroid dysfunction, etc.) should also be taken into account. In case an abnormality is detected, appropriate treatment must be performed.

## Data Availability

Not applicable.
